# Intracardiac Cement Embolism Following Vertebroplasty

**DOI:** 10.7759/cureus.87535

**Published:** 2025-07-08

**Authors:** Ali Hamade, Mohammad Darwish, Bilal Shoumar, Ali Sayyed, Claudette G Najjar, Malek Moussa

**Affiliations:** 1 Department of Cardiology, Lebanese University Faculty of Medical Sciences, Hadath, LBN; 2 Department of Radiology, Beirut Cardiac Institute, Beirut, LBN; 3 Department of Cardiothoracic Surgery, Beirut Cardiac Institute, Beirut, LBN; 4 Department of Cardiology, Beirut Cardiac Institute, Beirut, LBN

**Keywords:** cement extravasation, intracardiac cement embolism, multimodality cardiac imaging, right heart dysfunction, vertebroplasty complication

## Abstract

This case report presents a 70-year-old female patient who was diagnosed with an intracardiac cement embolism two months after a percutaneous vertebroplasty of her L4 vertebra. Although percutaneous vertebroplasty is generally considered a safe procedure, this case highlights the potential for serious complications. The patient was on oral vitamin K antagonist for the past 20 years for an unprovoked deep vein thrombosis and on statins for a chronic dyslipidemia. Although being mildly dyspneic after the operation, the patient presented to the emergency department after two months for a worsening dyspnea. Diagnostic imaging confirmed the presence of a large cement embolus fixed in the coronary sinus, associated with extensive tricuspid valve damage. An open-heart surgery for cement embolus removal and replacement of the valve was performed. This case underscores the importance of such complications for a rapid diagnosis and treatment.

## Introduction

Percutaneous vertebroplasty (PV) is a minimally invasive interventional procedure generally used to treat patients suffering from symptomatic spinal pain through the injection of polymethyl methacrylate (PMMA), also known as cement injection, into the vertebral body of concern to provide a structural support to the affected area and relief patients’ symptoms [1,2].

Therefore, it remains important to acknowledge that percutaneous vertebroplasty is not completely safe, like all other invasive medical procedures [3,4]. Adverse events associated with PV include spinal cord and thoracoabdominal organ damage, hemorrhage resulting from improper needle insertion [5,6], spinal cord or nerve root compression or injury, venous thrombosis, and cardiopulmonary cement embolization due to bone cement extravasation beyond the vertebral body [7,8].

Cement extravasation, or leakage of cement, is a frequent complication with an incidence rate ranging from 5% to 80% [9,10]. The vertebral body is rich in venous structures, particularly the internal and external vertebral venous plexuses. These plexuses form a valveless, low-pressure network that communicates freely with segmental veins and can even connect to the inferior vena cava and azygos system. Thus, a cortical breach into the basivertebral veins or a direct injury of the external or internal venous plexus along the needle tract can lead to cement extravasation into the adjacent veins. Factors that influence the passage of the cement inside or outside the vertebral body can be classified into three categories, noting the characteristics related to the bone and fracture, the properties of the cement, and the elements of injection technique, such as the volume, velocity, pressure, and needle positioning [11,12].

Intracardiac cement embolism (ICE) is a rare complication of percutaneous vertebroplasty (PV), primarily attributed to cement leakage. This occurs when polymethylmethacrylate infiltrates the paravertebral venous plexus, subsequently gaining access to the vertebral venous system, traveling through the right heart chambers, and ultimately lodging in the pulmonary arteries. The incidence of intracardiac cement embolization was reported to be 3.9% in 2019 [13] and increased to 4.6% in 2020 [14].

In most cases, ICE remains clinically silent (93% of cases) [13]. However, symptomatic ICE can present across a broad clinical spectrum, from mild chest pain caused by a small pulmonary embolism (PE) [15] to severe dyspnea and hemodynamic instability resulting from cardiac perforation and tamponade, potentially culminating in catastrophic outcomes such as cardiac arrest and death [8].

Currently, the treatment advocated in the majority of published studies involves surgical intervention, either open-heart surgery or minimally invasive techniques, for cement extraction, particularly in scenarios of extensive embolic burden, significant valvular injury, large emboli, obstructive or not, and with or without associated heart failure [16,17]. Conversely, a limited number of studies advocate for anticoagulant therapy in cases of small, asymptomatic intracardiac emboli, aiming to promote encapsulation and reduce the risk of thrombus formation.

## Case presentation

After obtaining informed consent, we report the case of a 70-year-old woman who presented with dyspnea and was subsequently diagnosed with new-onset severe tricuspid regurgitation, attributed to intracardiac cement embolization following percutaneous vertebroplasty.

Her past medical and surgical history is as follows: unprovoked axillary vein thrombosis 20 years ago, managed with acenocoumarol 2 mg daily, which she continues; familial hypercholesterolemia, treated with rosuvastatin 20 mg daily; percutaneous vertebroplasty, performed two months prior for L4 stress fracture; and no known allergies to food or medications.

She arrived at the emergency department with severe dyspnea on minimal exertion and orthopnea. Her symptoms began within 24 hours of the vertebroplasty; however, they were initially tolerable, and she did not notify hospital personnel. In the days following the procedure, her respiratory symptoms progressively worsened. One week prior to presentation, she noted increasing lower extremity swelling. At triage, the patient was found to be tachypneic and desaturated.

The vital signs on admission included the following: transcutaneous oxygen saturation (room air): 92% (normal range is typically >95%); automated blood pressure: 110/70 mmHg (acceptable range varies by individual, generally between 110/60 and 130/85 mmHg); and respiratory rate: 26 breaths per minute (normal adult range is 16-24 breaths per minute).

Physical examination and investigations revealed a systolic murmur, and bilateral nasal crackles were noted. In addition, +2 pitting edema of both lower limbs is observed. Chest X-ray showed bilateral infiltrates. The electrocardiogram (ECG) indicated sinus tachycardia, a right bundle branch block, and left axis deviation (the previous ECG is unavailable for comparison). Table 1 displays laboratory findings.

**Table 1:** Blood sample result CRP: C-reactive protein; TSH: thyroid stimulating hormone; INR: international normalized ratio; LDH: lactate dehydrogenase; SGPT: serum glutamate pyruvate transaminase

Blood test (normal range)	Result
White blood cell (4.5-11 × 10^9^/L)	4.82
CRP (0.3-1 mg/dL)	1.87
Creatinine (0.5-1.2 mg/dL)	0.87
TSH (0.45-4.22 mU/L)	0.66
Total serum protein (6-8 g/dL)	6.5
Albumin level (3.4-5 g/dL)	3.8
Partial prothrombin time (25-35 seconds)	20
INR (0.8-1.2)	1.55 (high)
Platelets count (150-400 platelets/uL)	150
Hemoglobin (12-16 g/dL)	13
LDH (180-240 U/L)	542
SGPT (7-56 U/L)	82 (high)
D-dimers (220-500 ng/mL)	930 (high)
Troponin (0.015-0.04 ng/mL)	0.7 (high)

Other findings revealed that electrolytes, calcium, magnesium, and phosphorus levels were within the normal range, and blood cultures were collected. An echocardiogram was done urgently, showing a dilated right ventricle and atrium, severe tricuspid regurgitation, and an elevated systolic pulmonary pressure reaching 55 mmHg. A solid, elongated structure originating near the coronary sinus was noted as well. The initial differential diagnosis showed PE, infective endocarditis (IE), acute coronary syndrome (ACS), and cardiac neoplasm.

Inflammatory markers were within normal range, and blood cultures were negative, making IE less likely. Repeated troponin testing after one hour was 0.5 ng/mL, which reduced suspicion for ACS. As the patient already had the indication for anticoagulation due to her history of venous thrombosis, she was admitted for further investigations, systemic therapeutic anticoagulation, and decongestive therapy.

**Figure 1:**
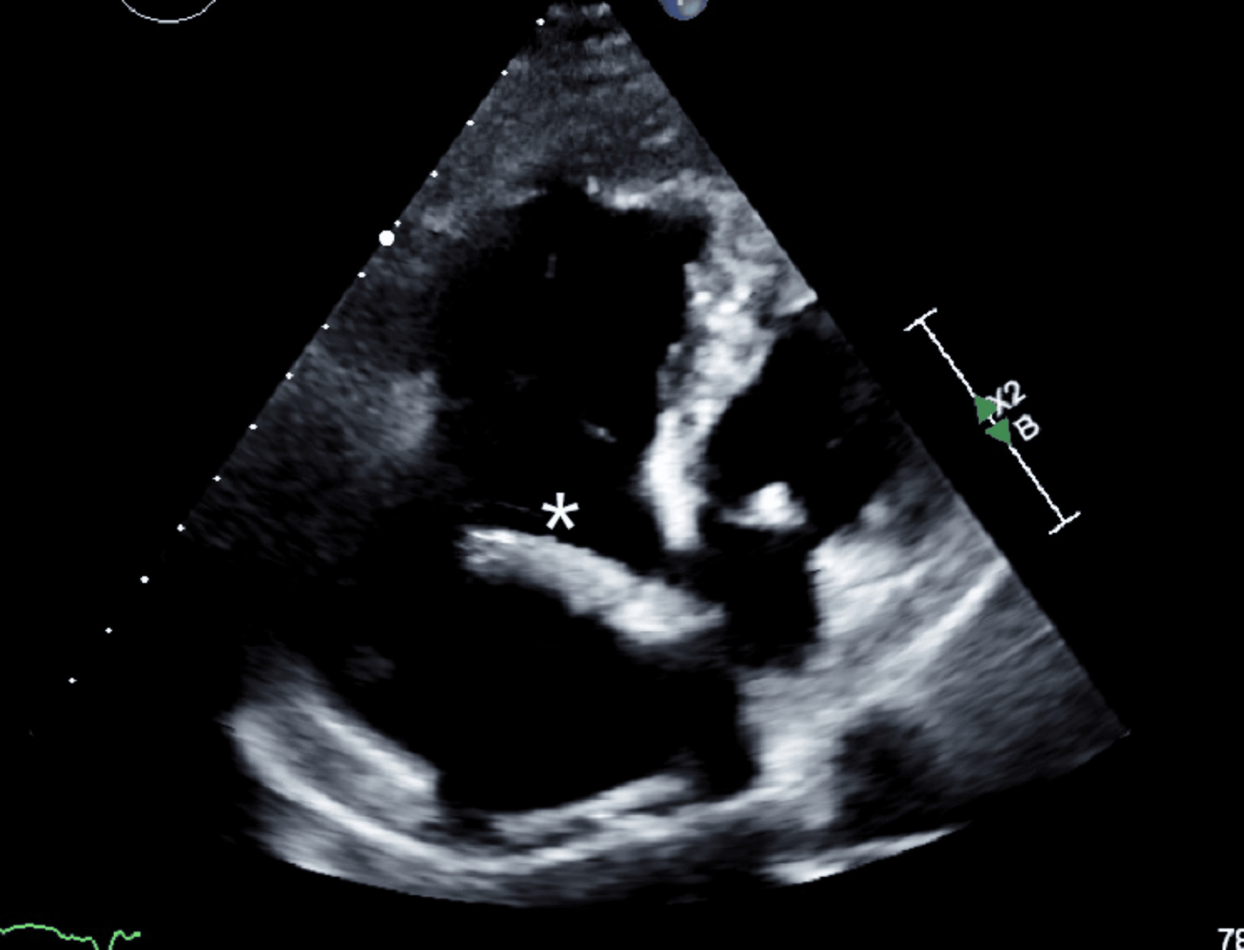
Transesophageal four-chamber view showing an elongated structure (asterisk) originating from the coronary sinus

**Figure 2:**
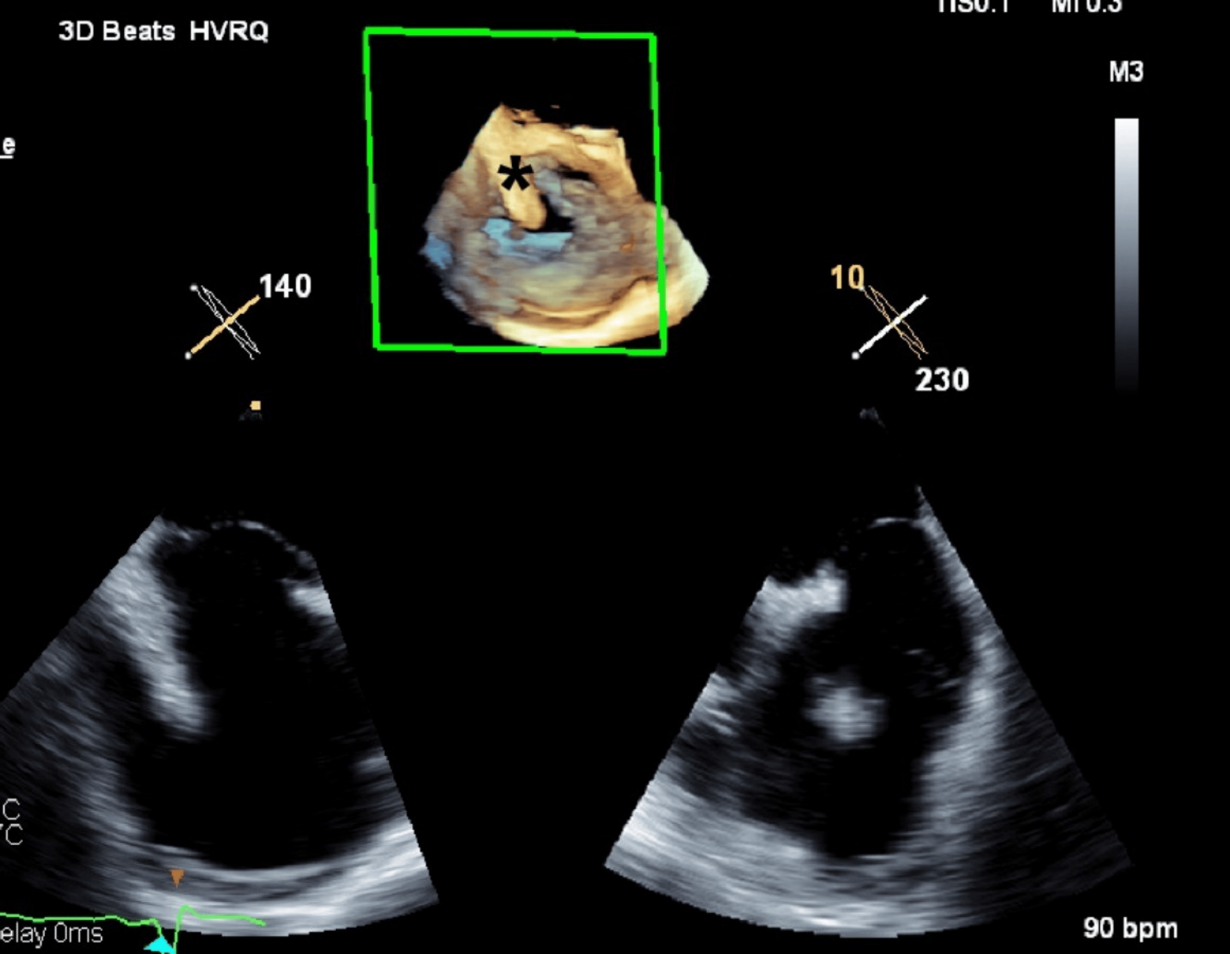
Transoesophageal echocardiogram cut showing the structure (asterisk) in 3D reconstruction 3D: three dimensional

**Figure 3:**
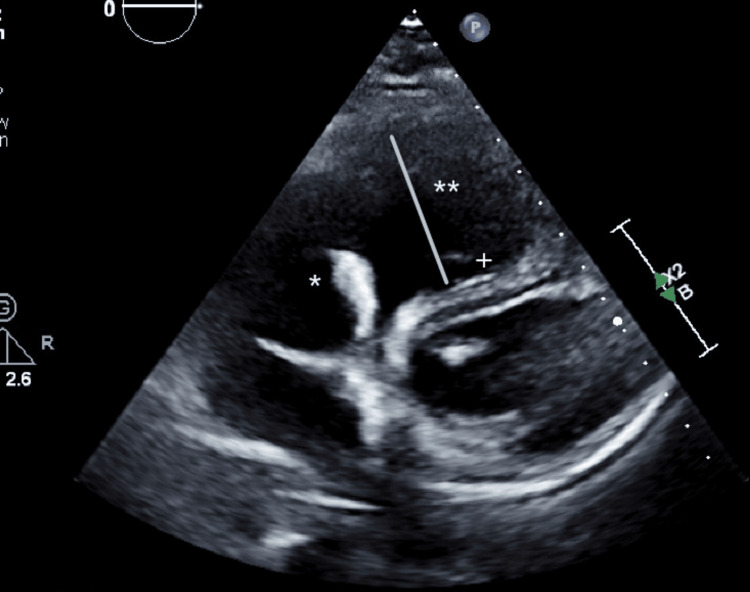
Transesophageal echocardiogram short-axis cut showing the abnormal structure (asterisk), the D-shaped right ventricle (double asterisks), and flattened septum (plus sign). The D-shaped right ventricle and flattened septum are findings found in right ventricle overload

**Figure 4:**
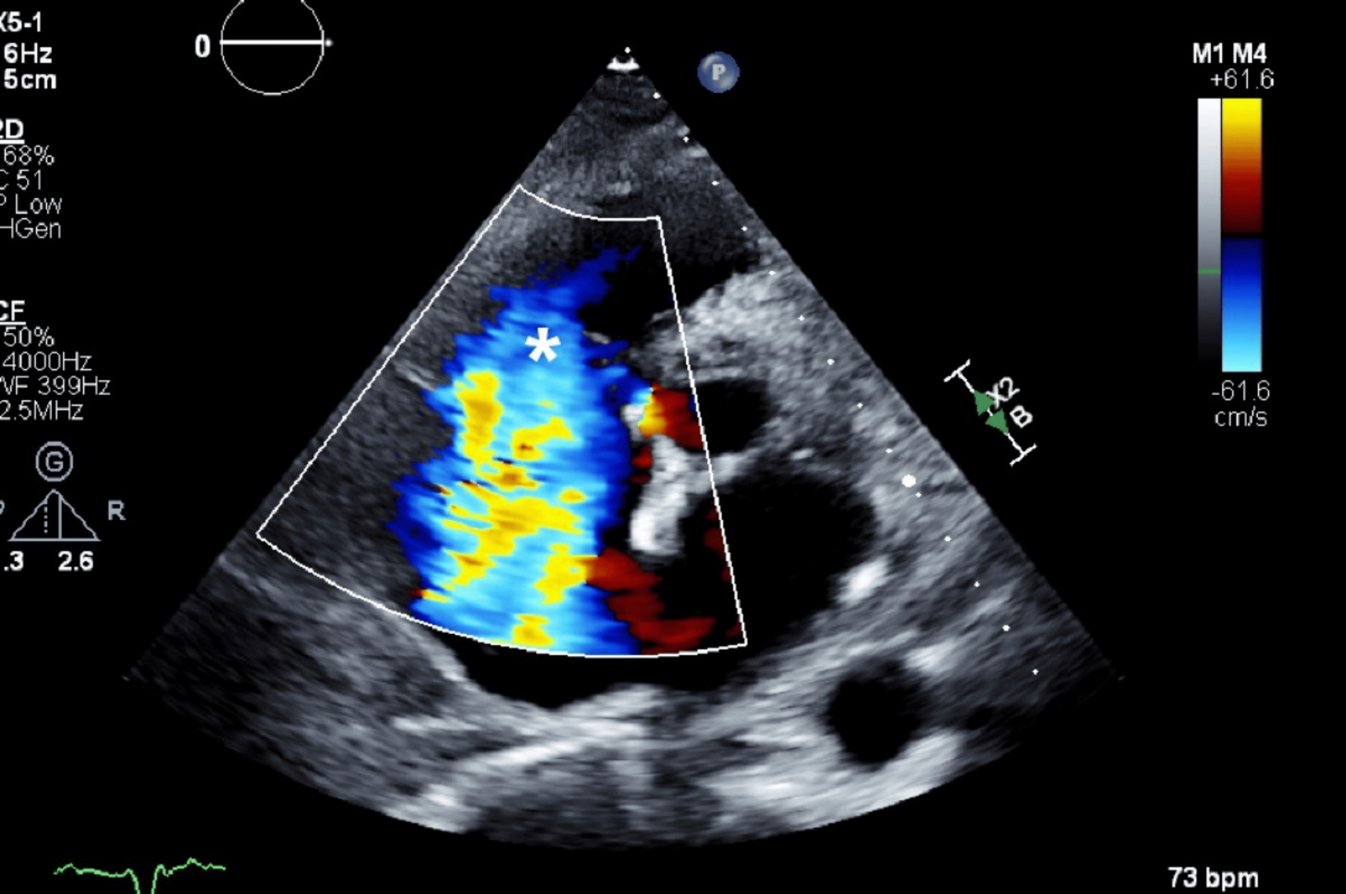
Transesophageal short-axis cut showing the huge central jet (asterisk) of severe tricuspid regurgitation

A transesophageal echocardiography (Figures 1, 2) was performed, revealing a markedly dilated right atrium containing a mobile mass measuring approximately 40 mm in length, originating from the coronary sinus. Additionally, the right ventricle appeared dilated (Figure 3) but demonstrated preserved systolic function, suggestive of right-sided volume overload. The tricuspid valve showed malcoaptation of the leaflets (Figure 4) during systole and annular dilation (normal range: <40 mm), resulting in severe tricuspid regurgitation. Mild pericardial effusion was also noted on the echographic assessment.

**Figure 5:**
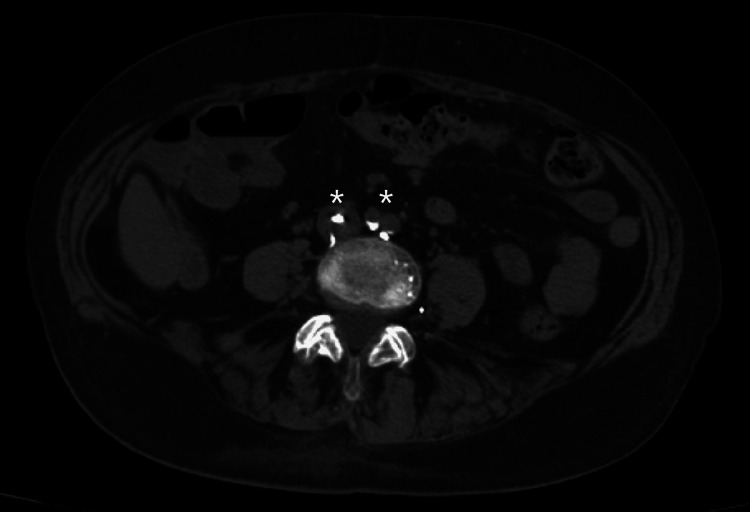
CT scan of the abdomen (axial cut) showing hyperdense emboli (asterisks) with the same density as the injected material in the repaired vertebra CT: computed tomography

**Figure 6:**
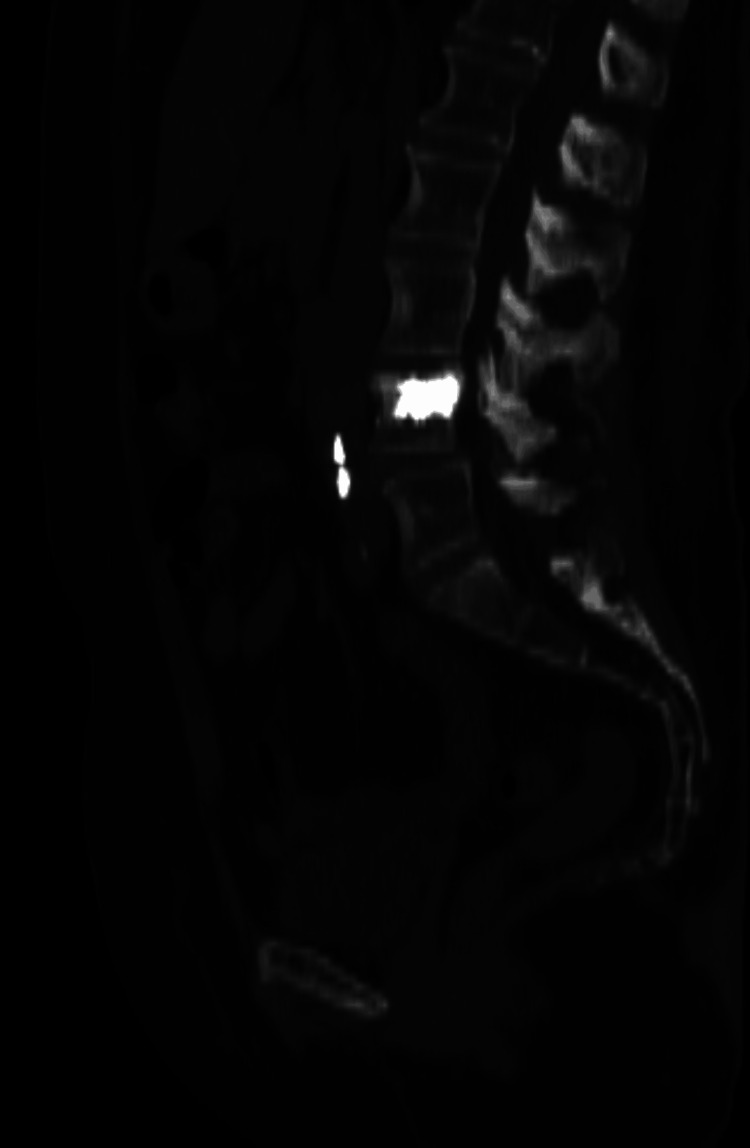
CT scan of the abdomen (sagittal cut) showing hyperdense emboli in the adjacent venous system with the same density as the injected material in the repaired vertebra CT: computed tomography

**Figure 7:**
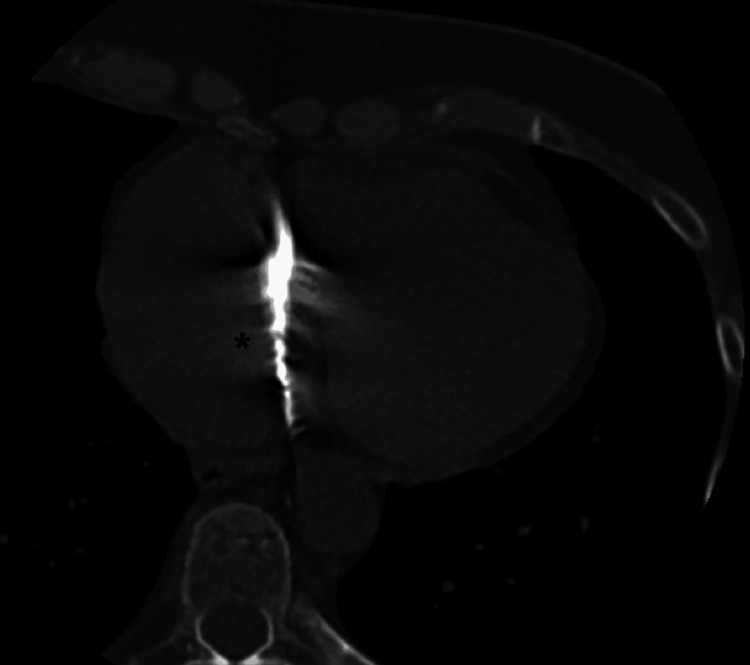
CT scan of the heart (coronal cut) showing the cardiac structure (asterisk) with the same density as the injected and infiltrated material CT: computed tomography

For further characterization of the mass and with the raised suspicion for cement embolization in this context , a computed tomography (CT) scan of the spine was done. Vascular infiltration of the injected product was detected near the repaired vertebra, showing the same density as the cardiac structure (Figures 5-7).

**Figure 8:**
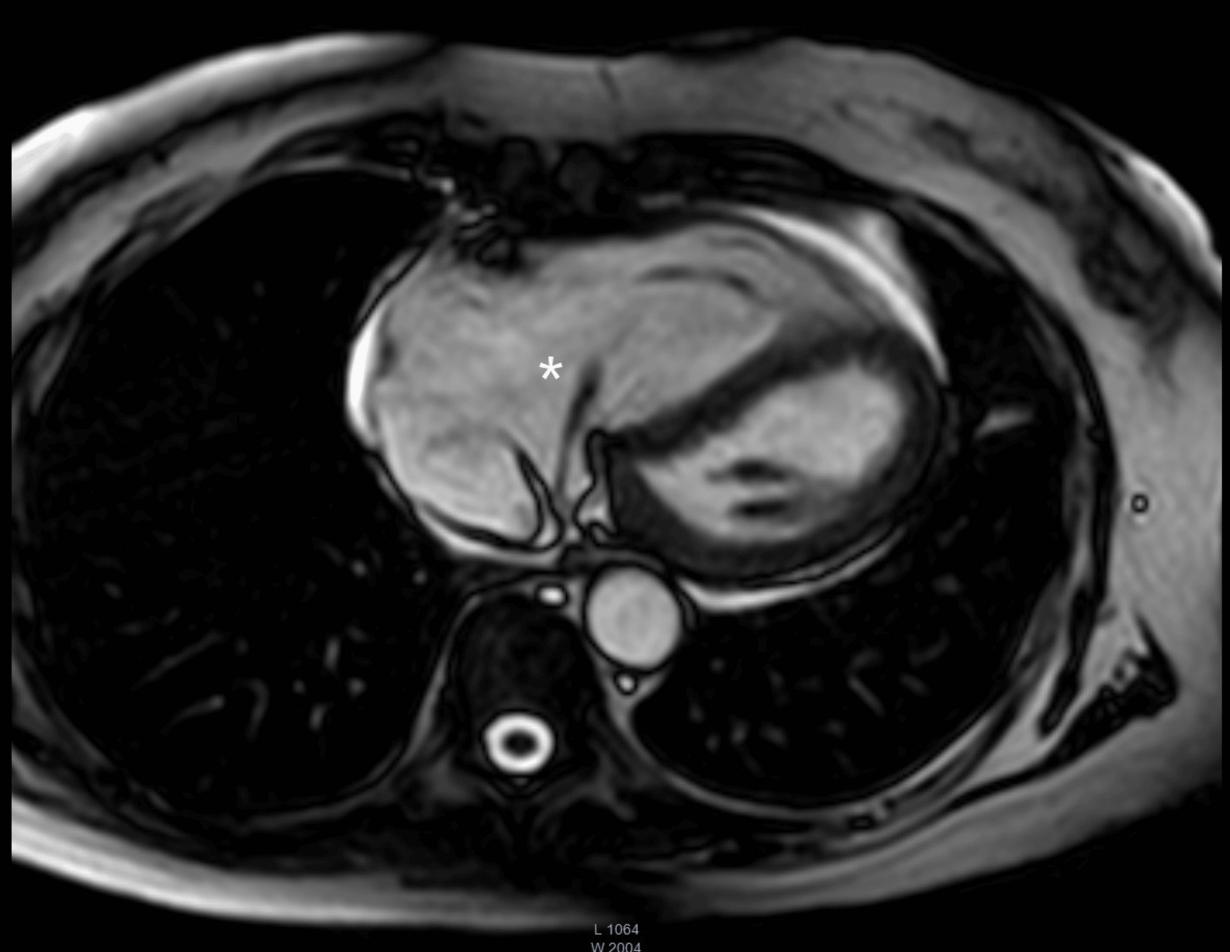
Cardiac MRI (coronal cut) showing the cardiac structure (asterisk) originating from the coronary sinus MRI: magnetic resonance imaging

**Figure 9:**
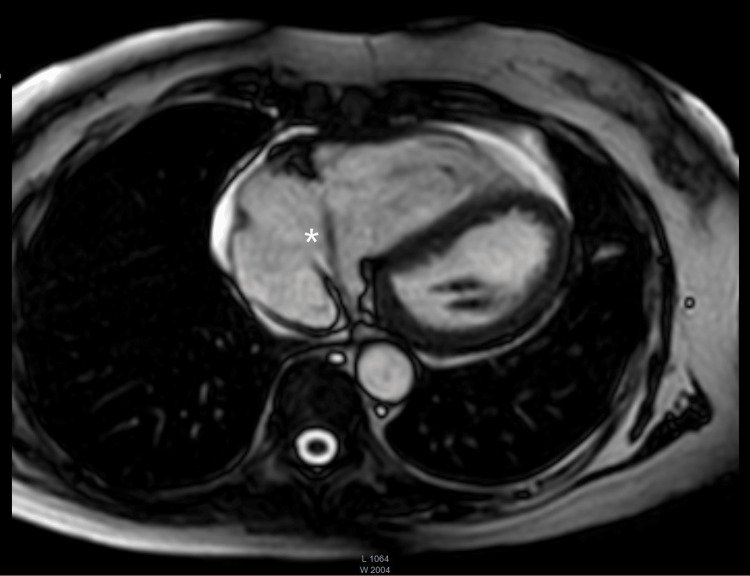
Another cardiac MRI (coronal cut) showing the cardiac structure (asterisk) originating from the coronary sinus MRI: magnetic resonance imaging

Additionally, a cardiac magnetic resonance imaging (MRI) scan showed significant dilation in the right atrium (25 cm², normal range <24 cm²), further highlighting the right heart overload due to the severe tricuspid regurgitation. Additionally, a right ventricular dilation was further confirmed, supported by the increased right-sided heart strain. A circumferential pericardial effusion was seen, with a 42 x 4 mm linear mobile mass originating from the coronary sinus into the right atrium, posing the differential diagnosis of cement embolism from vertebroplasty (Figures 8, 9).

Based on the results, a decision of surgical extraction was made, especially since the foreign body was remarkably large, coupled with tricuspid valve impairment. Prior to the procedure, a routine carotid Doppler ultrasound performed showed no abnormal findings, except for a mild thickness in the carotid intima-media (0.9 mm, normal range: 0.6-0.9 mm), indicating no significant stenosis or abnormal flow in the carotid arteries.

**Figure 10:**
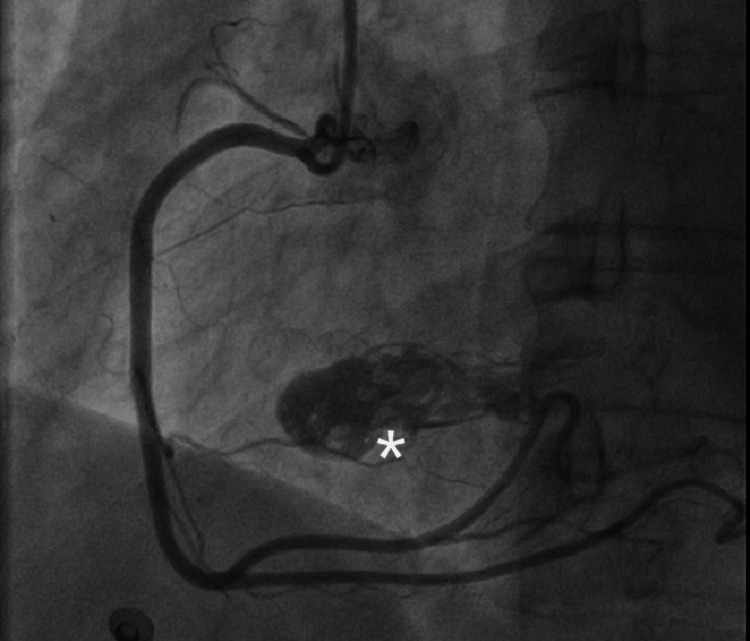
Coronary angiogram in the left anterior oblique view showing the abnormal elongated mass (asterisk)

**Figure 11:**
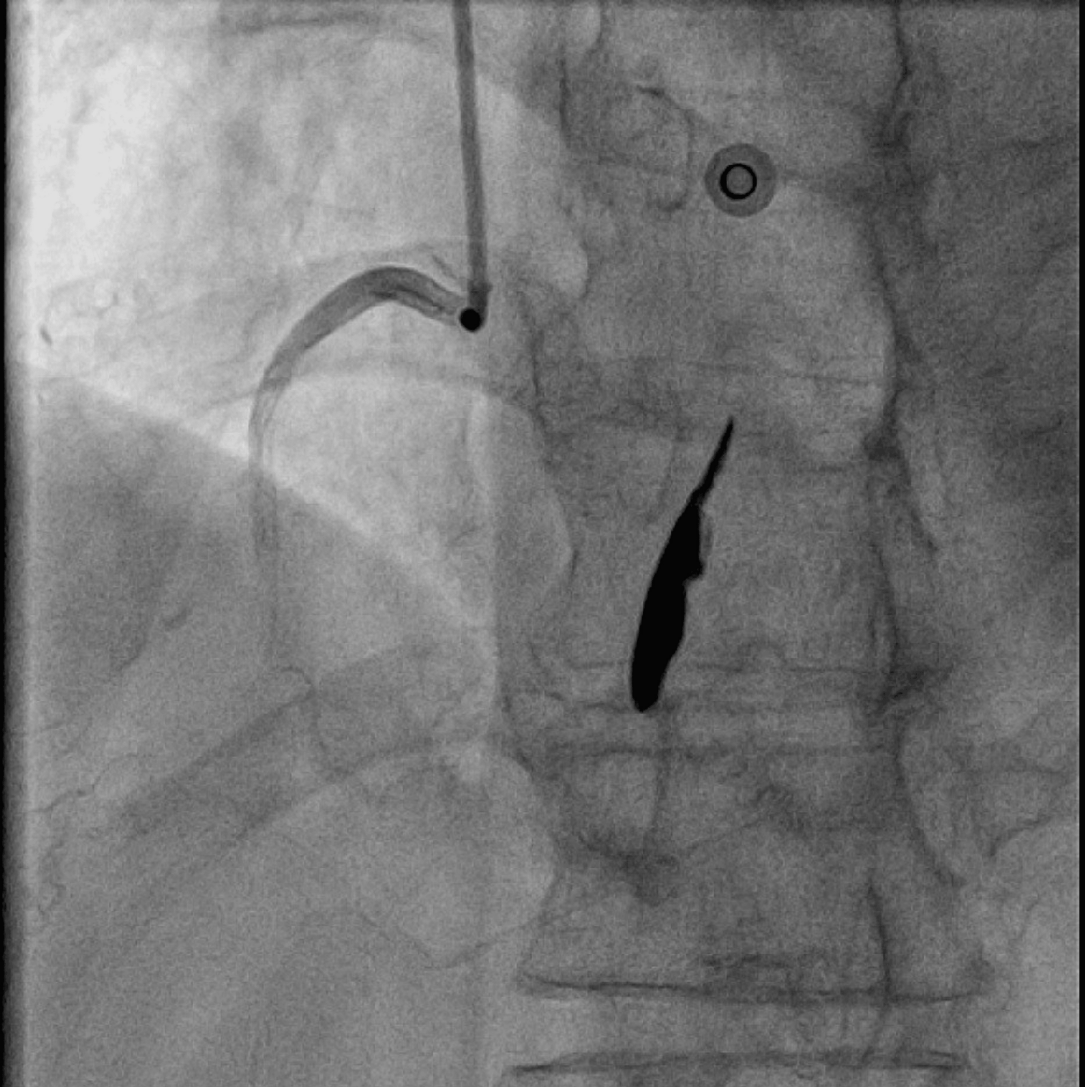
Coronary angiogram in the anteroposterior view showing the abnormal elongated mass

For coronary artery assessment, a coronary angiogram was performed. A mobile solid structure was observed in the right atrium during the right coronary artery injection in the left anterior oblique and the anteroposterior views (Figures 10, 11). The epicardial coronary arteries were free of disease.

A multidisciplinary team meeting was held, and the patient was scheduled for cardiac surgery to remove the foreign body and further explore the tricuspid valve. The patient underwent a median sternotomy with cardiopulmonary bypass. The cement was shown to be firmly adherent to the tricuspid valve leaflets. During the right atriotomy, the cement embolus was successfully dissected and extracted. Therefore, due to irreversible valve damage, a bioprosthetic tricuspid valve (Hancock 33 mm) was implanted, and the patent foramen ovale was closed to prevent paradoxical embolism. During the surgery, the patient maintained normal vital signs with an immediate return to normal rhythm upon aortic declamping and discontinuation of cardiopulmonary bypass. Drainage tubes were placed, pericardial and sternal tissues closed, and soft tissue repaired.

After the operation, the patient was transferred to the postcardiac surgery unit for observation. The recovery of the patient was unremarkable, with no significant signs or symptoms detected. She was treated with furosemide 40 mg intravenously daily and spironolactone 25 mg to decrease the right ventricular overload. In addition, therapeutic anticoagulation with heparin was initiated for thrombosis prevention in her newly implanted valve, and then switched to acenocoumarol 2 mg daily, as the patient was already on this regimen for unprovoked deep vein thrombosis. A postoperative cardiac echography was performed after five days of the surgery, showing a high effectiveness of the surgery, especially with the absence of any significant valvular dysfunction, and a normal function of the tricuspid bioprosthesis, along with normal functions of the right and left ventricles. The estimated systolic pulmonary artery pressure was 22 mmHg. Additionally, it was noted that the mild presurgical pericardial effusion had resolved, indicating a smooth recovery.

## Discussion

Since its introduction in 1987, percutaneous vertebroplasty has become a widely used surgical approach for treating fractures that cause spinal compression [18]. Despite the fact that ICE is often asymptomatic when a minimal amount of cement embolizes [19], it should be kept in mind that even small emboli may be lethal if embolization results in cardiac perforation. Symptoms associated with cement emboli are highly variable, and the presentation may be acute, subacute, or chronic. The most feared outcomes are cardiac perforation, acute valvular injury, and paradoxical cerebral embolism [20].

Tricuspid regurgitation may result from direct valvular injury, such as leaflet perforation, or from restricted leaflet motion due to cement deposits. In contrast, annular dilation and right ventricular enlargement, secondary to outflow tract obstruction, pulmonary valve stenosis, or a large pulmonary embolism, can lead to functional (secondary) tricuspid regurgitation [21].

Our patient presented with a progressive nontolerated dyspnea and desaturation, and was found to have severe tricuspid regurgitation and right heart overload requiring hospital admission and invasive intervention. 

Spinal radiographs are commonly used to detect perivertebral venous leakage. While large-volume emboli may be visible on conventional chest radiographs, these modalities have limitations in precisely localizing leakage sites due to interference from overlying anatomical structures. In contrast, chest CT is more sensitive for identifying small-volume, asymptomatic cement emboli. CT enables differentiation between injected, extravasated, and embolized material based on density comparisons [22,23].

Although data on the utility of cardiac MRI in this context are lacking, our imaging revealed significant right heart volume overload secondary to valvular dysfunction, along with detailed structural characterization of the right atrium.

Several approaches for the treatment of cardiac cement embolism have emerged over the years. Among these, we note the conservative treatment through the use of anticoagulants, as seen in the cases published by Xu et al. and Mills et al. [24,25]; the percutaneous retrieval, such as the cases published by Yang et al. [26]; and the open-heart surgery performed by Liu et al. and Krithika et al. [17,20]. 

In this case, the embolized cement fragment was large, mobile, and firmly adherent to the coronary sinus, and the tricuspid valve leaflets exhibited an irreversible structural damage. Given the high risk of fragmentation and procedural failure with percutaneous removal, combined with the patient’s favorable surgical risk profile and the need for tricuspid valve replacement, open-heart surgery was selected as the most appropriate intervention.

Furthermore, it is worth noting that the surgical procedure was successful, as the patient returned to her normal cardiac rhythm, with a normalization of the right ventricular size and function. Hence, the patient had an uneventful recovery, highlighting the effectiveness of the surgical approach in restoring the normal right heart function post-intracardiac cement embolism.

## Conclusions

Bone cement embolization is a well-recognized complication of percutaneous vertebroplasty. Its clinical manifestations can range widely, influenced by factors such as embolus size, location, and any associated valvular dysfunction. While some experts advocate for postoperative chest radiography as a surveillance measure, its sensitivity may be limited in comparison to CT or echocardiography.

It is essential for clinicians to maintain a high index of suspicion, as cardiopulmonary symptoms may present in a delayed fashion following vertebroplasty. Prompt recognition and timely intervention are critical for improving patient outcomes.
